# Are Perceived Effort Scales (OMNI-RES) Appropriate for Defining and Controlling Strength Training Intensity?

**DOI:** 10.3390/sports13020057

**Published:** 2025-02-17

**Authors:** José Luis Maté-Muñoz, Luis Maicas-Pérez, Iñigo Aparicio-García, Juan Hernández-Lougedo, Luis De Sousa-De Sousa, Mónica Hontoria-Galán, Francisco Hermosilla-Perona, Manuel Barba-Ruiz, Pablo García-Fernández, Juan Ramón Heredia-Elvar

**Affiliations:** 1 Faculty of Nursing, Physiotherapy and Podiatry, Complutense University of Madrid, 28040 Madrid, Spain; jmate03@ucm.es (J.L.M.-M.); luisdeso@ucm.es (L.D.S.-D.S.); 2 Department of Physical Activity and Sports Science, Alfonso X El Sabio University, 28691 Madrid, Spain; lmaicper@uax.es (L.M.-P.); iapargar@uax.es (I.A.-G.); mhontgal@uax.es (M.H.-G.); fperoher@uax.es (F.H.-P.); mruizbar@uax.es (M.B.-R.); jelvaher@uax.es (J.R.H.-E.); 3 Physiotherapy and Health Research Group (FYSA), Faculty of Health Sciences—HM Hospitals, Camilo José Cela University, 28692 Madrid, Spain; jlougedo@ucjc.edu; 4 Instituto de Investigación Sanitaria—HM Hospitales, 28692 Madrid, Spain; 5 Sport and Training Research Group, Department of Sports, Faculty of Physical Activity and Sport Sciences, Universidad Politécnica de Madrid (UPM), 28040 Madrid, Spain

**Keywords:** strength, sport performance, human performance, velocity, load, training, repetitions, fatigue

## Abstract

Background: One of the most significant challenges for exercise professionals in designing strength training programs is determining the intensity or effort level of each set performed. One of the most studied methodologies has been the use of Rate of Perceived Exertion (RPE) scales. This study aims to analyze the application of the OMNI-RES scale for monitoring training intensity across different relative loads and fatigue levels in various training protocols. Methods: In this cross-sectional study, participants completed nine exercise sessions, with one week separating each session. The first session involved a one-repetition maximum (1RM) test in the bench press (BP) to identify the load–velocity relationship. Subsequently, each participant randomly performed two maximum repetition (MNR) protocols at 60% and 90% of 1RM, and two protocols with a 30% velocity loss (VL) at 60% of 1RM and a 10% VL at 90% of 1RM. These sessions were repeated one week later. Results: significant differences were found between the four bench press protocols regarding the number of repetitions and the percentage of velocity loss per set (*p* < 0.001). However, the RPE of the MNR protocol at 60% of 1RM was significantly higher than the other protocols. Moreover, the RPE for the protocol at 60% of 1RM with a 30% VL was similar to that at 90% of 1RM with a 10% VL (*p* = 1.000). Post-exercise blood lactate concentrations, percentage VL at 1 m·s^−1^, and the effort index were significantly higher in the MNR protocol at 60% of 1RM compared to all other protocols (*p* < 0.001). Conclusions: The most important finding of this study is that the OMNI-RES scale may not be a reliable indicator of exercise intensity. This is because the highest values on the scale were observed at the lowest relative intensity (60% 1RM) during the maximum number of repetitions (MNR) protocol, corresponding to the maximum volume.

## 1. Introduction

An adequate definition and control of strength training variables are crucial for establishing accurate relationships between the applied stimuli and the outcomes achieved through training. Specifically, it seems that the load or level of effort imposed is primarily determined by the relative load (percentage of one-repetition maximum, %1RM) used and the degree of fatigue experienced during each set in strength training [1,2,3]. Over the past few years, research has focused on identifying the most appropriate and precise methods to define and control these variables [4,5,6,7,8,9,10].

One of the most significant and critical challenges for exercise professionals when designing strength training programs is determining the intensity or level of effort for each set performed [11]. In this context, intensity is commonly identified by the relative load (%1RM) associated with the resistance used or the magnitude of the absolute load (weight). Meanwhile, volume is determined by the total number of repetitions completed per set [1,7,8,12,13,14].

Both methods of defining and controlling the magnitude of the load seem to present numerous challenges and limitations, which may lead to significant disadvantages and errors when applied by exercise professionals [5,15,16,17]. Consequently, alternative approaches have been proposed to address these issues, offering greater precision by monitoring movement velocity during strength training. This method helps determine the relative load used (intensity) and the repetitions exerted (volume) [3,5,18,19]. Additionally, indicators such as the so-called “effort index” have been suggested to provide a more comprehensive understanding of the total load (intensity × volume) used in training [11,20].

This advancement has been achieved through the use of technology. However, as an alternative for exercise professionals without access to such technology, the use of scales for the Rate of Perceived Exertion (RPE) has been proposed for years and has been a focus of research since the development of the first scale by Borg [21,22,23,24]. This scale was initially based on the idea that complex mechanisms derived from metabolic, cognitive, and perceptual processes were responsible for producing a subjective sensation in response to a given stimulus. Borg’s original scale was designed to serve as an indicator of exercise intensity (primarily for endurance activities), using numerical values (initially 6–20, later the CR-10) and their correlation with criterion variables such as heart rate and, subsequently, others like VO_2max_ and blood lactate [25,26,27,28,29].

The use of these scales was controversial, leading to Robertson’s proposal of the OMNI-RES scale, specifically designed for strength training [30,31]. This scale features ten numerical values (with 0 representing “extremely easy” and 10 “extremely hard”) alongside pictograms to enhance participant understanding. The reliability and validity of these scales have been explored through various studies, using criterion variables such as total load lifted and blood lactate [30,31], heart rate, and electromyography values [32,33,34], as well as correlations with movement velocity [35,36,37].

However, certain limitations have been identified when these scales are used in research, particularly in their practical application. These scales are often employed to monitor the effort exerted [26,28,38], which aligns with their original definition. Nevertheless, as previously mentioned, it is essential to consider that the training load imposed by each exercise set is directly related to two fundamental variables: intensity (the effort required for the first repetition of the set, which correlates with a percentage of the individual’s maximum capacity) and volume (linked to the mechanical and metabolic stress imposed by the total repetitions in the set, which correlates with the decrease in achieved velocity). In other words, the assessment of effort should reflect the relationship between these two variables and, consequently, the level of fatigue that this relationship imposes.

The OMNI-RES scale has been proposed as a tool for defining training intensity [39,40]. This approach can be valid if appropriate teaching, familiarization, and education methods are applied, particularly when used to assess the effort required to complete the first repetition of a set with an absolute load. However, this method may be less sensitive than other indirect measures, such as effort characterization [19,41,42]. We can observe, for example, that some original studies on the validation of the scales³⁰ were conducted using analytical exercises at the same intensity level (with important limitations in the way it was determined), in which different numbers of repetitions (volume) were performed.

Using the scale to monitor intensity based on the number of repetitions completed or the number of repetitions in reserve may lead to inaccuracies [43,44,45,46,47]. Rather than reflecting intensity directly, it is more likely to indicate the level of metabolic stress caused by the effort. Metrics such as repetitions performed, repetitions in reserve (i.e., the number of potential repetitions left), and velocity loss during a set are all influenced by both intensity and volume; essentially, the degree of fatigue experienced [11,20].

This study examines the use of the RPE (OMNI-RES) scale to monitor training intensity, previously defined through the control of movement velocity, across different relative loads and levels of fatigue in various training sets.

Considering this, our team aims to analyze the application of the OMNI-RES scale for defining and controlling training intensity, linking this variable to mechanical and metabolic indicators, as well as the effort index achieved. This analysis will provide more precise insights into the utility of these scales in strength training programs, enabling professionals to make better-informed decisions regarding their use.

## 2. Materials and Methods

### 2.1. Study Design

In this cross-sectional study, participants completed nine exercise sessions, with one week separating each session. The first session involved a progressive load test in the bench press (BP) up to one-repetition maximum (1RM), aimed at identifying the load–velocity relationship for each participant. The subsequent eight sessions consisted of four different exercise protocols (Test), each repeated one week later (Retest). The four Test protocols were performed in random order for each participant. The week after the Test, the Retest was performed. In other words, there was one week between the Test and Retest for each protocol. The same BP technique as in the 1RM test was used in all cases.

The Tests involved performing a single set of BP exercises with different loads. During all exercise sessions, the mean propulsive velocity (MPV) of each repetition was measured (velocity values of the barbell during the propulsive phase, defined as the portion of the concentric phase where barbell acceleration was ≥9.81 m·s^−2^). All tests were conducted in the university’s training laboratory. Participants completed the nine exercise sessions on the same day of the week, within the same time window (±2 h) to control for circadian rhythms [48], and under the same environmental conditions (temperature between 18 and 22 °C and 40–55% humidity).

Participants underwent two familiarization sessions with the BP exercise one week before the study, separated by a 48 h interval.

### 2.2. Participants

Forty-six male physical activity and sport science students (22.61 ± 3.12 years, 77.78 ± 10.76 kg, 1.78 ± 0.07 m, and a body mass index of 24.67 ± 2.93 kg·m^−2^) participated in the present study. The participants were divided into three groups based on their relative strength ratio (RSR), which was obtained based on their 1RM strength/body mass ratio: a High-RSR group (RSR > 1.15, *n* = 10), a Medium-RSR group (0.95 ≤ RSR ≤ 1.15, *n* = 22), and a Low-RSR group (RSR < 0.95, *n* = 14). All participants were proficient in the technical execution of the BP exercise and did not have metabolic, orthopedic, or cardiorespiratory limitations that could affect their performance.

The GRANMO statistical calculator was used to estimate sample size, employing the standard deviation determined in a previous study (2.51) [49], a minimum difference of 1, an alpha error of 0.05, a power of 0.80, and a 5% loss-to-follow-up rate. Based on these parameters, a sample size of 43 participants per intervention group was calculated.

During the study, participants refrained from consuming dietary supplements, stimulants, or medications. They were instructed not to consume any food at least two hours before testing, with water being the only exception. Participants were also advised to avoid physical activity the day before each test. One week prior to testing, participants were informed of the research protocol and voluntarily signed informed consent forms. The university’s ethics committee approved the study protocol in accordance with the principles of [50].

### 2.3. Procedures

#### 2.3.1. RM Test

The protocol used was based on the study by [5], in which a progressive load test was performed in the BP exercise until reaching 1RM. This test determined the load–velocity relationship using the equation derived from the regression curve, calculated individually for each participant. Before the 1RM test, participants completed a warm-up consisting of 5 min of low-intensity running and 5 min of joint mobility exercises combined with dynamic stretches. This was followed by one set of 10 BP repetitions with a fixed load of 10 kg and another set of 5 BP repetitions with a fixed load of 20 kg.

The technical execution of the BP was as follows: participants lay in a supine position on a horizontal bench, with hips and knees flexed and feet placed on the bench. Their hands were positioned slightly wider than shoulder-width apart. The barbell was lowered in a controlled, slow manner to the chest, just above the intermammary line, and rested on the chest for 2 s. This pause enhanced repetition reliability by eliminating the rebound effect [51]. The concentric phase began after a verbal command, with an investigator timing the 2 s pause between the eccentric and concentric phases. The concentric phase was performed at maximum velocity, avoiding rebounds and ensuring that the shoulders and torso remained in contact with the bench.

#### 2.3.2. Maximum Number of Repetitions (MNR) Test

**Figure 1:**
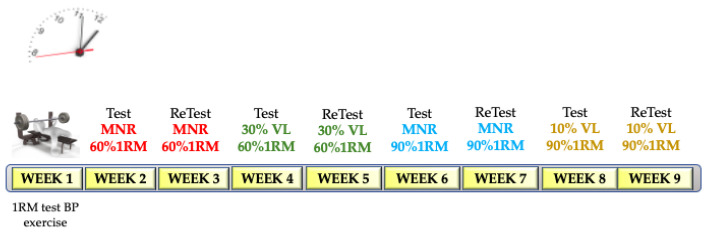
Example of a randomized protocol for one participant. Study design. 1RM = one-repetition maximum; BP = bench press; MNR = maximum number of repetitions; VL = velocity loss.

In two separate sessions, these tests consisted of performing a single set of bench presses (BPs) at the maximum number of repetitions (MNR) possible until muscle failure, using loads equivalent to 60% and 90% of the 1RM. The load was determined through the MPV obtained from the load–velocity relationship in the 1RM test for each participant as it has been demonstrated that there is a strong correlation between %1RM and MPV for this exercise [5]. Each participant’s absolute load (kg) was individually adjusted to match the velocity associated (±0.02 m·s^−1^) with the predicted %1RM for each session. The Retests were conducted one week after performing the MNR tests at 60% and 90% of the 1RM (Figure 1).

#### 2.3.3. Velocity Loss (VL) Test

Participants performed two sessions of single-set BP tests, completing repetitions until reaching a predetermined percentage of VL. For the 60% MPV load, repetitions were performed until a 30% loss in MPV was achieved, while for the 90% MPV load, repetitions continued until a 10% MPV loss was reached. These velocity losses were intended to be representative of a low mechanical and metabolic stress character according to previous studies [10]. Both sessions were repeated one week later (Figure 1).

#### 2.3.4. Blood Lactate Concentration

Capillary blood samples (5 μL) were collected from the fingertip of the index finger to determine blood lactate concentrations before the warm-up and 3 min after each test.

#### 2.3.5. OMNI-RES Scale

To measure the perceived effort of participants immediately after completing each of the sets in the four different protocols and their Retests, the OMNI-RES scale with ten numerical values (from 0 to 10) was used [30]. Participants were instructed on using the scale through validated procedures [52], which involved reading the instructions prior to each set and administering absolute loads corresponding to 60% and 90% of the 1RM. This allowed participants to relate the “sensations” they perceived during these loads to the values on the scale.

The instructions provided followed those established by Robertson [30], stating: “Use the pictograms to describe how your body feels during the exercise. You will now perform a set of exercises with external resistance for the upper body. Please look at the person at the bottom of the scale. If you feel like that person during the repetitions, your effort will be extremely easy. In this case, your response will be 0. Now look at the person at the top of the scale. If you feel like that person during the repetitions, your effort will be extremely hard. Therefore, your response will be 10. If you feel that your effort is somewhere between these values, select the corresponding number. Remember, there are no wrong answers. All values are valid. Your response may change as the load lifted changes. Use both the pictograms and the text to decide your final response”.

#### 2.3.6. Mechanical Fatigue Test

To quantify the mechanical fatigue induced by all strength protocols, the percentage change in MPV was measured pre- and post-exercise using an individual load lifted at ~1 m·s^−1^ MPV (MPV at 1 m·s^−1^ Tests) in BP. Participants performed lifts with progressively heavier weights to determine the individual load corresponding to 1 m·s^−1^ until this velocity was achieved. The protocol began with the barbell (10 kg), and the load was increased in increments of 1.25–5 kg, with participants performing three repetitions per load and resting for three min between loads.

Sánchez-Medina and González-Badillo [19] selected the velocity of 1 m·s^−1^ because it represents a sufficiently high velocity achieved with moderate loads (approximately 45–50% 1RM in BP). This load is relatively easy to lift, well-tolerated, and effectively demonstrates the relationship between load and velocity. The mean MPV of the three repetitions performed before the exercise was compared with the mean MPV of the three repetitions performed after the exercise [19]. All repetitions were performed at maximum velocity.

#### 2.3.7. Measurement Equipment

Lactate measurements were performed using a portable lactate analyzer that had been previously validated and calibrated (Lactate Pro 2 LT-1710, Arkray Factory Inc., KDK Corporation, Siga, Japan) [53,54]. Additionally, the tests were conducted using a Smith machine (Matrix, Chácara Alvorada, Brazil) with a guided barbell. Weight plates of 20, 10, 5, 2.5, and 1.25 kg (Matrix) were used. In this setup, both ends of the barbell were fixed, allowing only vertical movement of the bar.

A previously validated optoelectronic device was used to estimate the velocity of each repetition during the various tests [55]. This device operated at a sampling frequency of 500 Hz (Velowin v.1.7.232, Instrumentos y Tecnología Deportiva; Murcia, Spain). The software used for MPV analysis automatically calculated the results using internally generated algorithms (Velowin v.1.7.232).

### 2.4. Statistical Analysis

The Shapiro–Wilk test was used to verify the normality of the variables. Second-order polynomials were employed to establish the load–velocity relationship for each participant in the progressive load test up to 1RM. To analyze the different variables in the four BP exercise protocols in the total group of participants, a one-factor repeated-measures ANOVA was performed. To analyze the relationship between the different variables in the four BP exercise protocols (MNR 60%, 1RM; MNR 90%, 1RM; 30% VL 60%, 1RM; 10% VL 90%, 1RM), with the subgroups based on strength level (High RSR, Medium RSR, and Low RSR), a multifactor repeated-measures ANOVA was performed, with Mauchly’s sphericity test applied. When the sphericity hypothesis was rejected, the univariate F-statistic was adjusted using the Greenhouse–Geisser correction factor. The effect of the BP Protocols × Strength Level interaction was also analyzed, applying Bonferroni’s post hoc index for the pairwise comparison.

Moreover, the effect size was determined using partial eta squared (ηp^2^), categorizing the magnitude of the difference as trivial (ηp^2^ ≤ 0.01), small (0.01 ≤ ηp^2^ < 0.06), moderate (0.06 ≤ ηp^2^ < 0.14), or large (ηp^2^ ≥ 0.14) [56], alongside the statistical power (SP) of the data. Intrasubject variability was examined using the standard error of measurement (SEM), calculated as the square root of the mean square error term in a repeated-measures ANOVA [57].

Additionally, Bland–Altman’s systematic bias ± random error and the coefficient of variation (CV), expressed as a percentage of the mean results, were used [58]. All data were expressed as means, standard deviations (SD), 95% confidence intervals (CI), and minimum–maximum ranges (Min–Max). The significance level was set at *p* < 0.05. All statistical analyses were conducted using SPSS version 25.0 (SPSS, Chicago, IL, USA).

## 3. Results

In the progressive load test up to 1RM, data were collected for MPV and load (kg) at 1RM, as well as at 60% and 90% of the MPV at 1RM (Table 1). These data were analyzed for the total group of participants and for the subgroups classified by their RSR. The subgroups consisted of 10 participants with High RSR (1.39 ± 0.16), 22 participants with Medium RSR (1.05 ± 0.58), and 14 participants with Low RSR (0.74 ± 0.10).

Analyzing the protocols that reach the MNR, it was observed that the protocol at 60% of 1RM reached an MPV of 0.16 m·s^−1^, while the protocol at 90% of 1RM reached an MPV of 0.18 m·s^−1^, values compatible with the MPV at 1RM (Table 2). Additionally, the protocols with a 30% and 10% VL reached an average velocity loss of approximately 28% and 9%, respectively.

Significant differences were found between the four bench press protocols regarding the number of repetitions and the percentage of velocity loss per set (*p* < 0.001), with differences observed across all pairwise comparisons (*p* < 0.05) (Table 2). However, the RPE of the MNR protocol at 60% of 1RM was significantly higher than the other protocols. Moreover, the RPE for the protocol at 60% of 1RM with a 30% MPV loss was similar to that at 90% of 1RM with a 10% VL (*p* = 1.000).

Post-exercise blood lactate concentrations ([La]), percentage VL at 1 m·s^−1^, and the effort index were significantly higher in the MNR protocol at 60% of 1RM compared to all other protocols (*p* < 0.001). In contrast, the 60% of 1RM protocol with a 30% VL showed no significant differences in post-exercise [La], percentage VL at 1 m·s^−1^, or effort index when compared to the MNR protocol at 90% of 1RM (*p* > 0.05). However, both protocols differed significantly from the 90% of 1RM protocol with a 10% VL (*p* > 0.05).

**Figure 2:**
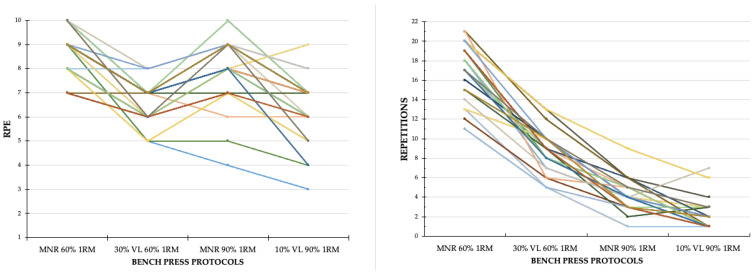
Spaghetti plots representing the RPE and repetitions of the four bench press protocols. Each colored line represents each of the subjects and the scores obtained in both their RPE and the number of repetitions performed in each of the four protocols.

Figure 2 shows the RPE values together with the number of repetitions performed for the four BP exercise protocols, showing that the protocol with the highest RPE and the highest number of repetitions performed is the MNR protocol at 60% of 1RM.

Analyzing the variables by strength level (Table 3), significant differences were found in the number of repetitions for the BP Protocol factor, the Strength Level factor, and their interaction (BP Protocol × Strength Level, *p* < 0.05). Pairwise comparisons using Bonferroni’s post hoc adjustment confirmed that the number of repetitions varied depending on the BP protocol at each strength level (*p* < 0.05), with this being the protocol with the most repetitions in the MNR protocol at 60% 1RM. Specifically:

- The High-RSR group performed significantly more repetitions than the Low-RSR group in the MNR protocol at 60% of 1RM and the protocol with a 30% VL at 60% of 1RM (*p* = 0.001, *p* < 0.001, respectively).
- In the MNR protocol at 90% of 1RM, the High-RSR group performed significantly more repetitions than the Medium- and Low-RSR groups (*p* = 0.032, *p* < 0.001).
- In the Medium-RSR group, a significant difference was found only when compared to the Low-RSR group in the protocol with a 30% VL at 60% of 1RM (*p* < 0.001).

Regarding RPE, significant differences were found for the BP Protocol factor, the Strength Level factor, and the BP Protocol × Strength Level interaction (*p* < 0.05). RPE values were significantly higher in the MNR protocol at 60% of 1RM for the High-RSR group compared to the other protocols (*p* < 0.05). In the Medium- and Low-RSR groups, RPE values were higher compared to the protocol with a 30% VL at 60% of 1RM and the protocol with a 10% VL at 90% of 1RM (*p* < 0.05) but not when compared to the MNR protocol at 90% of 1RM (*p* > 0.05). When analyzing differences between strength groups within each protocol, significant differences were found only in the 10% VL at 90% of 1RM and the MNR protocol at 90% of 1RM (*p* < 0.05). For the remaining protocols, RPE values were similar (*p* > 0.05).

For post-exercise lactate and the velocity loss percentage at 1 m·s^−1^, significant differences were found for the BP Protocol factor, the Strength Level factor, and the BP Protocol × Strength Level interaction (*p* < 0.05). Post-exercise lactate was significantly higher in the MNR protocol at 60% of 1RM across all strength levels (*p* < 0.05), except for the Low-RSR group, where similar values were observed between the MNR protocol at 60% of 1RM and the protocol with 30% VL at 60% of 1RM (*p* > 0.05).

Additionally, the MNR protocol at 60% of 1RM resulted in a significantly greater velocity loss at 1 m·s^−1^ compared to all other protocols across all strength groups (*p* < 0.05). Moreover, velocity loss was lower in the High-RSR group compared to the Medium-RSR group in the MNR protocol at 90% of 1RM (*p* < 0.05) and compared to the Low-RSR group in the protocol with a 10% VL at 90% of 1RM (*p* < 0.05).

The MNR protocol at 60% of 1RM exhibited the highest effort index compared to all other protocols across all three strength level groups (*p* < 0.05).

Table 4 presents the test–retest variability. No significant differences (*p* > 0.05) were found in the MNR protocol at 60% of 1RM between T2 and T3 for any variable except for the percentage of MPV at 1 m·s^−1^. Significant differences were observed in the protocol, with a 30% VL at 60% of 1RM in the number of repetitions (*p* = 0.021) and RPE (*p* = 0.038). For the MNR protocol at 90% of 1RM between T2 and T3, significant differences were found only in MPVrep Last (*p* = 0.002) and the percentage of velocity loss during the set (*p* = 0.034). Finally, in the protocol with a 10% VL at 90% of 1RM, significant differences were observed in RPE (*p* = 0.007), the percentage of velocity loss during the set (*p* = 0.002), post-exercise lactate (*p* < 0.001), and the effort index (*p* = 0.046).

It is also worth noting that RPE values showed relatively good consistency across the four exercise protocols, with a coefficient of variation (CV) ranging from 5% to 11%. Post-exercise lactate values showed acceptable variability in the two MNR protocols (9–13%) and MPVrep Best across all protocols except for the 10% VL at 90% of 1RM (6–10%). Acceptable values were also observed for the number of repetitions in the two MNR protocols and the 30% VL protocol at 60% of 1RM (7–22%), as well as for MPVrep Last (11–19%), the percentage of velocity loss during the set (5–23%), and the effort index (8–25%).

Within-individual test–retest variability was further examined using Bland–Altman. A systematic bias was identified for all analyzed variables in all RSR groups, with the lowest bias observed in the High-RSR group (Table 5). This systematic bias remained low when the overall participant group was analyzed, with the exception of the effort index variable (Table 5).

## 4. Discussion

Rate of Perceived Exertion scales (OMNI-RES) are commonly used to define and monitor strength training intensity [39,45,59,60,61]. In this study, we analyzed the comparison of two relative intensities (60% 1RM and 90% 1RM) where there were two different effort levels at each intensity: one where the maximum number of repetitions is performed and another where different training volumes are used. These scales should be able to clearly distinguish an intensity associated with these varying degrees of effort based on participants’ perceptions.

In the different studies in the literature [30,35,36,44], the scales have been used considering only indicators of relative intensity (%1RM, velocity, power), total load (volume × intensity, repetitions in reserve), or metabolic stress, being used to characterize the degree of effort reached after training, and subsequently used in some studies to define the intensity of the training [45].

The most important finding of this study is that the OMNI-RES scale may not be a reliable indicator of exercise intensity. The highest values on the scale were observed at the lowest relative intensity (60% 1RM) during the maximum number of repetitions protocol, corresponding to the maximum volume. These higher OMNI-RES values were associated with greater fatigue, as they coincided with the protocol involving the highest number of repetitions, a greater percentage of velocity loss within the set, higher metabolic stress, and a higher effort index.

Despite participants being instructed according to protocols described in the scientific literature [30,31] and having performed a progressive load test, which familiarized them with the full range of relative loads associated with their individualized absolute values, the results contradict the potential value of these scales for assessing training intensity [45,60,61]. A possible explanation for these discrepancies is that many studies analyze the number of repetitions performed at a fixed intensity, typically estimated from the 1RM value. This approach may inherently lack precision as current evidence indicates that controlling intensity in this manner is less accurate. Furthermore, determining training volumes based on a uniform number of repetitions could compare substantially different degrees of effort [4,11,42,46]. In our study, we attempted to ensure some standardization of the independent variables by controlling the velocity of the repetitions and the velocity loss within each set [3,4,5,11].

When the group performed at maximum volume for each relative intensity, the greatest differences were observed in the scale values associated with mechanical and metabolic stress. In this case, the perceived effort was higher at a lower intensity (60% compared to 90%). Under these conditions, the number of repetitions required to reach maximum fatigue was significantly higher, as were the percentage of velocity loss, lactate levels, and changes in velocity at the 1 m·s^−1^ load. These results indicate that metabolic stress and fatigue were at their highest levels.

This is further confirmed by the analysis of the effort index, which was significantly higher at 60% intensity with the MNR protocol despite similar perceived effort values on the OMNI-RES scale. In this case, participants rated the efforts as “hard.” Evidently, the factor influencing this rating varied: in some instances, it was the high relative intensity (90%), while in others, it was the significantly lower relative intensity (60%) combined with a maximum volume that required a large number of repetitions, leading to greater metabolic stress. This increased response in mechanical and metabolic stress levels at a high volume of work has been corroborated in numerous studies [4,19,42,62].

All these differences appear to be independent of the subjects’ strength levels. In other words, even though the degree of effort was similar across groups (as determined by velocity loss), stronger subjects (High RSR) completed more repetitions to reach the same velocity loss. This difference was statistically significant between groups for all protocols except for the 10% VL at 90% of 1RM.

However, the perceived effort recorded on the scales did not vary between groups (there was only the lowest RPE in the High RSR in the two protocols at 90% of 1RM). In most cases, perceived effort was highest when subjects reached their maximum possible repetitions, rather than at lower effort levels. Therefore, these scales may be useful to represent fatigue levels rather than exercise intensity.

It is possible that in similar cases, participants cannot rate the efforts on the scale with any other value than the one found in our study, even though it should be clearly different in favor of the higher intensity. The challenge of accurately determining training intensity, heavily influenced by volume, is further complicated by findings from recent studies [46,47]. These studies show that a significant percentage of participants may underestimate the prescribed or intended load for exercises. This underestimation arises from substantial variability in the number of repetitions that can be performed at the same relative intensity, with many athletes exceeding the expected repetition range [46,47].

The findings regarding effort ratings on the scale are consistent across all groups, regardless of relative strength level (RSR). This is observed even when the number of repetitions varies, as previously mentioned, for a similar velocity loss within a set. These results highlight the significant fatigue experienced at lower intensities when achieving the highest volume. The mechanical and physiological findings indicate that, in this case, the substantial degree of fatigue appears to hinder an accurate rating of intensity, with participants perceiving the effort as “hard.”

This finding raises questions about the very definition of terms used in effort perception scales, which have historically employed terms such as “exertion” and, later, “tired” [30,31], often applied across diverse populations [63]. Participants seem to find it easier to define “effort” in terms of “fatigue”, particularly when high-volume loads are involved. This may present even greater challenges when the goal is not to evaluate the effort performed but rather to prescribe a specific intensity for a strength exercise, as proposed in some studies [45].

The present study was carried out using a single exercise and a sample with experience in strength training and different levels of performance; however, future studies should analyze other exercises and perhaps the use of the scales in subjects highly specialized in strength (such as weightlifters or powerlifters), as well as controlling for variables that may affect subjectivity in the perception of effort, such as stress level, motivation, mood, or even dietary control. In addition, due to the fact that in this study there is a reduction in statistical power when comparing groups with different levels of strength, studies with a larger number of participants are needed to reinforce these positions.

## 5. Conclusions

Perceived effort scales in strength training (OMNI-RES) may be more closely related to the degree of fatigue than to the intensity achieved during a single set of exercises. This association aligns with their origin and connection to certain physiological variables. Intensity could potentially be evaluated if a single repetition were performed with the absolute load, but this would require participants to undergo training across the entire range up to their 1RM, which may be inadvisable in many cases.

On the other hand, velocity-based monitoring offers an accessible and precise method for determining intensity. A single repetition performed at the maximum possible velocity, or even estimating the effort level, would be a safer and more advisable approach in most scenarios.

Therefore, our findings underscore the challenges of relying solely on perceived effort scales for prescribing exercise intensity. These scales fail to reliably distinguish intensity after completion and are heavily influenced by fatigue-related stress, which is more closely linked to volume. This highlights the need for integrating additional objective measures, such as velocity loss or physiological markers, to complement perceived effort scales in strength training.

Future studies should analyze the sensitivity of OMNI-RES scales across the full range of relative intensities, including greater variability in effort levels at each intensity. This would also allow for a deeper understanding of their utility in assessing the degree of fatigue through these scales.

## Figures and Tables

**Table 1:** Data recorded from the 1RM test.

		MPV 1RM (m·s^−1^)	MPV 60% (m·s^−1^)	MPV 90% (m·s^−1^)	MPV at 1 m·s^−1^ (% 1RM)	KG 1RM (kg)	KG 60% 1RM (kg)	KG 90% 1RM (kg)
	M ± SD	0.17 ± 0.06	0.75 ± 0.08	0.34 ± 0.05	39.78 ± 7.67	80.00 ± 22.56	48.13 ± 12.56	73.26 ± 18.34
Total Group (*n* = 46)	95% CI	0.16–0.19	0.73–0.77	0.32–0.35	37.50–−42.06	73.30–86.70	44.40–51.86	67.80–78.72
	Min–Max	0.07–0.29	0.62–0.92	0.25–0.43	25–55	43–138	26–76	42–121
	CV	35%	11%	15%	19%	28%	26%	25%
	M ± SD	0.11 ± 0.03	0.66 ± 0.03	0.29 ± 0.04	32.00 ± 6.33	107.60 ± 26.05	61.80 ± 14.47	93.80 ± 23.28
High RSR (*n* = 10)	95% CI	0.09–0.14	0.64–0.69	0.26–0.32	27.50–36.52	89.00–126.23	51.45–72.15	77.15–110.45
	Min–Max	0.07–0.15	0.62–0.69	0.26–0.37	25–40	73–138	41–76	63–121
	CV	27%	5%	14%	20%	24%	23%	25%
	M ± SD	0.18 ± 0.05	0.76 ± 0.06	0.34 ± 0.05	40.91 ± 4.79	81.55 ± 7.06	49.45 ± 6.27	75.00 ± 6.43
Medium RSR (*n* = 22)	95% CI	0.15–0.20	0.73–0.78	0.32–0.36	38.79–43.03	78.42–84.67	46.67–52.23	72.15–77.85
	Min–Max	0.09–0.26	0.66–0.89	0.25–0.43	35–50	71–96	38–60	65–88
	CV	28%	8%	15%	12%	9%	29%	5%
	M ± SD	0.21 ± 0.05	0.81 ± 0.06	0.37 ± 0.03	43.57 ± 8.64	57.28 ± 9.66	36.29 ± 6.27	55.86 ± 8.49
Low RSR (*n* = 14)	95% CI	0.18–0.24	0.77–0.85	0.36–0.39	38.58–48.56	52.28–63.44	32.67–39.91	50.95–60.76
	Min–Max	0.14–0.29	0.69–0.92	0.32–0.41	25–55	43–71	26–46	42–66
	CV	24%	5%	8%	5%	17%	17%	15%

MPV = Mean propulsive velocity; 1RM = one-repetition maximum; RSR = Relative strength ratio, defined as 1RM divided by body mass; M = mean ± SD = standard deviation; CI = confidence interval; Min–Max = lowest value—highest value; CV = Coefficient of variation; KG = kilograms.

**Table 2:** Data of the different variables of the four bench press protocols for the total group of participants (*n* = 46).

Variable	MNR 60% 1RM (M ± SD, 95% CI, CV)	30% VL 60% 1RM (M ± SD, 95% CI, CV)	MNR 90% 1RM (M ± SD, 95% CI, CV)	10% VL 90% 1RM (M ± SD, 95% CI, CV)	*η_p_* ^2^ *SP*	*p*
Repetitions (nº)	16.83 ± 3.01 *	8.83 ± 2.18	4.17 ± 1.62	2.22 ± 1.62	0.935 1.000	<0.001
	15.93–17.72	8.18–9.48	3.69–4.66	1.73–2.70		
	18%	25%	39%	73%		
RPE	8.70 ± 0.96 $ † ‡	6.57 ± 0.83 £	8.04 ± 1.28 #	6.48 ± 1.30	0.611 1.000	<0.001
	8.41–8.98	6.32–6.81	7.66–8.42	6.09–6.86		
	11%	13%	16%	20%		
MPV_rep_ Best (m·s^−1^)	0.73 ± 0.06 † ‡	0.75 ± 0.06 £ &	0.35 ± 0.04	0.37 ± 0.14	0.880 1.000	<0.001
	0.15–0.20	0.15–0.20	0.15–0.20	0.15–0.20		
	8%	8%	11%	38%		
MPV_rep_ Last (m·s^−1^)	0.16 ± 0.05 $ ‡	0.54 ± 0.07 £ &	0.18 ± 0.05 #	0.34 ± 0.14	0.968 1.000	<0.001
	0.14–0.17	0.51–0.56	0.16–0.19	0.30–0.38		
	31%	13%	28%	28%		
% loss MPV Set	−78.48 ± 7.13 *	−27.63 ± 8.39	−49.01 ± 15.61	−8.61 ± 10.94	0.895 1.000	<0.001
	−80.60–−76.37	−30.12–−25.14	−53.65–−44.38	−11.86–−5.37		
	9%	30%	32%	127%		
Lactate PRE (mmol·L^−1^)	1.57 ± 0.44 $	1.80 ± 0.41	1.75 ± 0.44	1.69 ± 0.32	0.066 0.677	0.033
	1.44–1.70	1.68–1.92	1.60–1.87	1.59–1.78		
	28%	23%	25%	19%		
Lactate POST (mmol·L^−1^)	6.28 ± 1.60 $ † ‡	4.84 ± 1.52 &	4.43 ± 1.46 #	3.57 ± 1.26	0.470 1.000	<0.001
	5.81–6.76	4.39–5.29	3.99–4.86	3.20–3.94		
	25%	31%	33%	35%		
MPV at 1 m·s^−1^ PRE (m·s^−1^)	1.02 ± 0.03	1.01 ± 0.03	1.02 ± 0.03	1.02 ± 0.03	0.028 0.309	0.276
	1.01–1.03	1.00–1.02	1.01–1.03	1.01–1.03		
	3%	3%	3%	3%		
MPV at 1 m·s^−1^ POST (m·s^−1^)	0.57 ± 0.13 $ † ‡	0.84 ± 0.10 &	0.83 ± 0.11 #	0.92 ± 0.11	0.717 1.000	<0.001
	0.52–0.60	0.81–0.88	0.80–0.87	0.89–0.96		
	23%	12%	13%	12%		
% loss MPV at 1 m·s^−1^ (m·s^−1^)	−45.40 ± 13.10 $ † ‡	−16.33 ± 10.27 &	−18.32 ± 10.72 #	−9.53 ± 10.68	0.716 1.000	<0.001
	−49.29–−41.51	−19.38–−13.28	−21.50–−15.13	−12.70–−6.36		
	29%	63%	59%	112%		
Effort Index	57.05 ± 7.23 $ † ‡	20.55 ± 6.58 &	17.32 ± 6.41 #	3.30 ± 4.60	0.937 1.000	<0.001
	54.85–59.24	18.54–22.55	15.37–19.27	1.97–4.76		
	13%	32%	37%	137%		

MPV = Mean Propulsive Velocity; MNR = maximum number of repetitions; VL = Velocity Loss; RPE = Rating of Perceived Effort; MPV_rep_ Best = Mean propulsive velocity attained at the best repetition; MPV_rep_ Last = Mean propulsive velocity attained at the last repetition; PRE = pre-exercise, POST = post-exercise; M = mean ± SD = standard deviation; CI = confidence intervals; CV = Coefficient of variation; η_p_^2^ = partial eta-squared; SP = statistical power; *p* = level of significance. * = significant difference between all sets (*p* < 0.05). $ = significant difference between set 1 and set 2 (*p* < 0.05). † = significant difference between set 1 and set 3 (*p* < 0.05). ‡ = significant difference between set 1 and set 4 (*p* < 0.05). £ = significant difference between set 2 and set 3 (*p* < 0.05). & = significant difference between set 2 and set 4 (*p* < 0.05). # = significant difference between set 3 and set 4 (*p* < 0.05).

**Table 3:** Data of the different variables of the four bench press protocols for the three levels of strength.

Variables	Level of Strength	MNR 60% 1RM (M ± SD, 95% CI)	30% VL 60% 1RM (M ± SD, 95% CI)	MNR 90% 1RM (M ± SD, 95% CI)	10% VL 90% 1RM (M ± SD, 95% CI)	*p* Time *η_p_*^2^ *SP*	*p* Group *η_p_*^2^ *SP*	*p* Group × Time *η_p_*^2^ *SP*
Repetitions (nº)	High RSR (*n* = 10)	19.00 ± 1.76 * ¶	10.40 ± 1.96 ¶	5.60 ± 1.96 ¥ ¶	2.20 ± 2.04	<0.001 0.939 1.000	<0.001 0.393 0.997	0.012 0.145 1.000
		17.74–20.26	9.00–11.80	4.20–7.00	0.74–3.66			
	Medium RSR (*n* = 22)	17.09 ± 2.74 *	9.45 ± 1.65	4.18 ± 1.30	2.45 ± 0.91			
		15.88–18.31	8.72–10.19 §	3.61–4.76	2.05–2.86			
	Low RSR (*n* = 14)	14.86 ± 3.01 *	6.71 ± 1.44	3.14 ± 1.03	1.86 ± 2.18			
		17.74–20.26	9.00–11.80	4.20–7.00	0.74–3.66			
RPE	High RSR (*n* = 10)	8.40 ± 0.84 $ † ‡	6.20 ± 1.03	6.80 ± 1.81 # ¥ ¶	5.60 ± 1.59 ¥	<0.001 0.606 1.000	0.005 0.222 0.866	0.036 0.098 0.791
		7.80–9.00	5.46–6.94	5.50–8.10	4.47–6.73			
	Medium RSR (*n* = 22)	8.91 ± 0.92 $ ‡	6.55 ± 0.67 £	8.55 ± 0.80 #	6.82 ± 0.96			
		8.50–9.32	6.25–6.84	8.19–8.90	6.39–7.24			
	Low RSR (*n* = 14)	8.57 ± 1.09 $ ‡	6.86 ± 0.86 £	8.14 ± 0.86 #	6.57 ± 1.34			
		7.94–9.20	6.36–7.36	7.64–8.64	5.80–7.35			
MPV_rep_ Best (m·s^−1^)	High RSR (*n* = 10)	0.73 ± 0.03 † ‡	0.74 ± 0.05 £ &	0.32 ± 0.02 ¥ ¶	0.43 ± 0.23	<0.001 0.876 1.000	0.189 0.075 0.344	0.164 0.075 0.453
		0.71–0.75	0.70–0.78	0.30–0.33	0.27–0.60			
	Medium RSR (*n* = 22)	0.73 ± 0.07 † ‡	0.73 ± 0.06 £ & §	0.35 ± 0.03	0.34 ± 0.05			
		0.70–0.76	0.70–0.75	0.34–0.36	0.32–0.37			
	Low RSR (*n* = 14)	0.73 ± 0.08 † ‡	0.78 ± 0.04 £ &	0.37 ± 0.04	0.38 ± 0.16			
		0.69–0.78	0.76–0.80	0.35–0.39	0.29–0.47			
MPV_rep_ Last (m·s^−1^)	High RSR (*n* = 10)	0.14 ± 0.05 $ ‡	0.55 ± 0.03 £ &	0.16 ± 0.01 #	0.42 ± 0.24 ¥	<0.001 0.839 1.000	0.086 0.108 0.492	0.055 0.104 0.655
		0.11–0.18	0.53–0.58	0.15–0.17	0.25–0.59			
	Medium RSR (*n* = 22)	0.15 ± 0.05 $ ‡	0.52 ± 0.07 £ &	0.18 ± 0.05 #	0.29 ± 0.05			
		0.13–0.17	0.49–0.55	0.16–0.20	0.27–0.32			
	Low RSR (*n* = 14)	0.18 ± 0.06 $ ‡	0.55 ± 0.10 £ &	0.18 ± 0.06 #	0.36 ± 0.11			
		0.14–0.21	0.50–0.61	0.15–0.22	0.30–0.42			
% loss MPV Set	High RSR (*n* = 10)	−80.42 ± 6.54 *	−24.55 ± 4.07	−49.28 ± 3.06	−3.92 ± 5.58 ¥	<0.001 0.898 1.000	0.239 0.064 0.299	0.081 0.087 0.647
		−85.10–−75.74	−27.47–−21.64	−51.47–−47.10	−7.91–0.08			
	Medium RSR (*n* = 22)	−79.13 ± 7.04 *	−28.00 ± 7.16	−48.77 ± 14.74	−14.51 ± 11.98 §			
		−82.25–−76.01	−31.13–−24.79	−55.30–−42.23	−19.82–−9.20			
	Low RSR (*n* = 14)	−79.08 ± 7.53 *	−29.30 ± 11.79	−49.20 ± 22.05	−2.70 ± 6.87			
		−80.42–−71.73	−36.11–−22.50	−61.93–−36.48	−6.67–1.26			
Lactate POST (mmol·L^−1^)	High RSR (*n* = 10)	7.84 ± 1.53 $ † ‡ ¥ ¶	5.82 ± 1.76 & ¶	5.16 ± 1.31 #	3.26 ± 1.32	<0.001 0.516 1.000	0.013 0.184 0.771	0.004 0.134 0.928
		6.74–8.94	4.56–7.08	4.23–6.10	2.32–4.20			
	Medium RSR (*n* = 22)	6.21 ± 1.43 $ † ‡	4.76 ± 1.17 &	4.27 ± 1.38	3.26 ± 1.32			
		5.58–6.84	4.25–5.28	3.66–4.88	3.23–4.21			
	Low RSR (*n* = 14)	5.29 ± 1.02 † ‡	4.26 ± 1.60	4.14 ± 1.61	3.56 ± 1.48			
		4.70–5.87	3.33–5.18	3.21–5.07	2.70–4.41			
% loss MPV at 1 m·s^−1^ (m·s^−1^)	High RSR (*n* = 10)	−34.63 ± 12.33 $ † ‡ ¶	−14.23 ± 10.64	−9.40 ± 6.75 ¥	−1.26 ± 16.67 ¶	<0.001 0.729 1.000	<0.001 0.319 0.979	0.005 0.148 0.907
		−43.46–−25.81	−21.84–−6.62	−14.23–−4.58	−13.18–−10.67			
	Medium RSR (*n* = 22)	−44.64 ± 6.94 $ † ‡ §	−18.27 ± 11.47	−23.67 ± 10.73 #	−10.33 ± 6.32			
		−47.71–−41.56	−23.36–−13.18	−28.42–−18.91	−13.13–−7.53			
	Low RSR (*n* = 14)	−54.28 ± 15.36 $ † ‡	−14.79 ± 7.86	−16.28 ± 8.15	−14.18 ± 7.81			
		−63.15–−45.41	−19.33–−10.25	−20.96–−11.58	−18.69–−9.67			
Effort Index	High RSR (*n* = 10)	58.80 ± 4.54 $ † ‡	18.20 ± 4.02 &	15.60 ± 1.71 #	1.20 ± 1.69 ¥	<0.001 0.938 1.000	0.528 0.031 0.151	0.182 0.068 0.566
		55.56–62.05	15.32–21.08	14.38–16.83	0.01–2.41			
	Medium RSR (*n* = 22)	56.80 ± 7.74 $ † ‡	20.10 ± 5.24 &	17.30 ± 5.98 #	5.40 ± 4.55			
		53.18–60.42	17.65–22.55	14.50–20.10	3.27–7.53			
	Low RSR (*n* = 14)	56.14 ± 8.22 $ † ‡	22.86 ± 9.04 &	18.57 ± 8.80 #	2.00 ± 5.08			
		51.40–60.89	17.64–28.08	13.49–23.65	4.94–0.94			

RSR = Relative strength ratio, defined as 1RM divided by body mass; MPV = Mean Propulsive Velocity; MNR = maximum number of repetitions; VL = Velocity Loss; RPE = Rating of Perceived Effort; MPV_rep_ Best = Mean propulsive velocity attained at the best repetition; MPV_rep_ Last = Mean propulsive velocity attained at the last repetition; POST = post-exercise; M = mean ± SD = standard deviation; CI = confidence intervals; Min–Max = lowest value–highest value; CV = Coefficient of variation; η_p_^2^ = partial eta-squared; SP = statistical power; *p* = level of significance. * = significant difference between all sets (*p* < 0.05). $ = significant difference between set 1 and set 2 (*p* < 0.05). † = significant difference between set 1 and set 3 (*p* < 0.05). ‡ = significant difference between set 1 and set 4 (*p* < 0.05). £ = significant difference between set 2 and set 3 (*p* < 0.05). & = significant difference between set 2 and set 4 (*p* < 0.05). # = significant difference between set 3 and set 4 (*p* < 0.05). ¥ = significant difference between High RSR and Medium RSR (*p* < 0.05). ¶ = significant difference between High RSR and Low RSR (*p* < 0.05). § = significant difference between Medium RSR and Low RSR (*p* < 0.05).

**Table 4:** Intrasubject variability in the variables of the four bench press protocols of the 46 participants of the study.

	MNR 60% 1RM				30% VL 60% 1RM				MNR 90% 1RM				10% VL 90% 1RM			
(*n* = 46)	Test	Retest	SEM	CV	Test	Retest	SEM	CV	Test	Retest	SEM	CV	Test	Retest	SEM	CV
Repetitions	16.83 ± 3.01	16.87 ± 3.23	1.20	7%	8.83 ± 2.18	7.91 ± 3.36 *	1.82	22%	4.17 ± 1.62	4.39 ± 1.97	0.92	21%	2.22 ± 1.62	1.74 ± 1.58	1.33	67%
RPE	8.70 ± 0.96	8.57 ± 1.03	0.44	5%	6.57 ± 0.83	6.26 ± 1.24 *	0.68	11%	8.04 ± 1.28	7.78 ± 1.11	0.71	9%	6.48 ± 1.30	6.09 ± 1.36 *	0.66	10%
MPV_rep_ Best	0.73 ± 0.06	0.75 ± 0.05	0.04	6%	0.75 ± 0.06	0.76 ± 0.11	0.08	10%	0.35 ± 0.04	0.34 ± 0.04	0.03	9%	0.37 ± 0.14	0.36 ± 0.09	0.12	34%
MPV_rep_ Last	0.16 ± 0.05	0.17 ± 0.05	0.03	19%	0.54 ± 0.07	0.56 ± 0.07	0.06	11%	0.18 ± 0.05	0.16 ± 0.05 *	0.03	19%	0.34 ± 0.14	0.34 ± 0.06	0.11	32%
% loss MPV Set	−78.48 ± 7.13	−78.11 ± 6.40	3.93	5%	−27.63 ± 8.39	−25.67 ± 7.12	6.13	23%	−49.01 ± 15.61	−53.00 ± 14.87 *	8.77	17%	−8.61 ± 10.94	−3.06 ± 6.33 *	8.31	142%
Lactate POST	6.28 ± 1.60	6.07 ± 1.47	0.82	13%	4.84 ± 1.52	4.80 ± 1.89	1.58	33%	4.43 ± 1.46	4.53 ± 1.37	0.42	9%	3.57 ± 1.26	2.98 ± 0.83 *	0.72	22%
% loss MPV at 1 m·s^−1^	−45.40 ± 13.10	−43.40 ± 12.51 *	4.64	10%	−16.33 ± 10.27	−16.49 ± 7.52	8.33	51%	−18.32 ± 10.72	−18.90 ± 9.24	6.43	35%	−9.53 ± 10.68	−8.73 ± 10.29	7.23	79%
Effort Index	57.05 ± 7.23	57.91 ± 4.34	4.31	8%	20.55 ± 6.58	19.96 ± 5.79	4.87	24%	17.32 ± 6.41	18.30 ± 6.27	4.52	25%	3.30 ± 4.51	1.48 ± 4.15 *	4.27	179%

SEM = Standard error of measurement; MPV = Mean propulsive velocity; MNR = maximum number of repetitions; VL = Velocity Loss; RPE = Rating of Perceived Effort; MPV_rep_ Best = Mean propulsive velocity attained during the best repetition; MPV_rep_ Last = Mean propulsive velocity attained during the last repetition; POST = post-exercise. Data expressed as mean ± standard deviation. CV = Coefficient of variation. * = significant difference (*p* < 0.05).

**Table 5:** Bland–Altman of intra-individual variability (Test–Retest) of the number of repetitions, RPE, MPV_rep_ Best, MPV_rep_ Last, lactate post-exercise, and effort index on two different days according to strength.

	MNR 60% 1RM			30% VL 60% 1RM			MNR 90% 1RM			10% VL 90% 1RM		
	Systematic Bias	Random Error	CI (95%)	Systematic Bias	Random Error	CI (95%)	Systematic Bias	Random Error	CI (95%)	Systematic Bias	Random Error	CI (95%)
Repetitions	0.04	1.70	3.44 to −3.35	−0.91	2.58	4.25 to −6.07	0.22	1.30	2.81 to −2.38	−0.48	1.88	3.29 to −4.24
RPE	−0.13	0.62	1.11 to −1.37	−0.30	0.96	1.62 to −2.23	−0.26	1.00	1.74 to −2.26	−0.39	0.93	1.47 to −2.25
MPV_rep_ Best	0.02	0.06	0.14 to −0.10	0.01	0.11	0.23 to −0.20	0.01	0.05	0.09 to −0.11	−0.02	0.17	0.33 to −0.36
MPV_rep_ Last	0.01	0.04	0.10 to −0.08	0.02	0.10	0.20 to −0.16	0.02	0.03	0.05 to −0.08	0.001	0.15	0.31 to −0.30
Lactate POST	0.21	1.15	2.10 to −2.52	−0.04	2.23	4.43 to −4.50	0.10	0.59	1.28 to −1.08	0.59	1.02	1.46 to −2.63
Effort Index	−4.08	16.39	28.71 to −36.87	0.65	6.89	14.44 to −13.13	−1.09	6.39	11.69 to −13.86	1.83	6.03	13.90 to −10.24

MPV = Mean propulsive velocity; MNR = maximum number of repetitions; VL = Velocity Loss; Rep = repetitions; MPV_rep_ Best = Mean propulsive velocity attained during the best repetition; MPV_rep_ Last = Mean propulsive velocity attained during the last repetition. Data expressed as mean ± standard deviation. CI = confidence interval.

## Data Availability

Data are contained within the article.
